# Factors Affecting Post-Challenge Survival of *Flavobacterium psychrophilum* in Susceptible Rainbow Trout from the Literature

**DOI:** 10.3390/pathogens11111318

**Published:** 2022-11-10

**Authors:** Brian W. Avila, Kathryn P. Huyvaert, Dana L. Winkelman, Eric R. Fetherman

**Affiliations:** 1 Colorado Cooperative Fish and Wildlife Research Unit, Colorado State University, Fort Collins, CO 80523, USA; 2 Department of Fish, Wildlife, and Conservation Biology, Colorado State University, Fort Collins, CO 80523, USA; 3 Department of Veterinary Microbiology and Pathology, Washington State University, Pullman, WA 99164, USA; 4 U.S. Geological Survey, Colorado Cooperative Fish and Wildlife Research Unit, Colorado State University, Fort Collins, CO 80523, USA; 5 Colorado Parks and Wildlife, 317 West Prospect Road, Fort Collins, CO 80526, USA

**Keywords:** *Flavobacterium psychrophilum*, challenge experiment, mortality, Bayesian analysis

## Abstract

Infectious bacterial pathogens are a concern for aquaculture as estimates suggest that billions of US dollars are lost annually in aquaculture due to disease. One of the most prevalent salmonid pathogens is the bacterium *Flavobacterium psychrophilum* that causes bacterial coldwater disease. We reviewed the published *F. psychrophilum* literature and conducted a Bayesian analysis to examine large-scale patterns in rainbow trout (*Oncorhynchus mykiss*) mortality associated with laboratory challenge. We incorporated factors that were common across a majority of the laboratory exposure studies and these included bacterial dose, culture time, exposure method, bacterial isolate, experimental duration, and fish weight. The comparison showed that injection as the exposure method produced higher mortality than bath immersion, bacterial isolates differed in their effect on mortality, and bacterial dose has an interactive effect with fish weight and exposure method. Our comparison allows for inference on factors affecting rainbow trout mortality due to exposure to *F. psychrophilum* and suggests avenues to further optimize research protocols to better reach study goals.

## 1. Introduction

Diseases are a major concern in the production, management, and conservation of fish and wildlife populations and can contribute to the decline and extinction of wild and domesticated animal populations [1,2,3,4,5,6,7]. Infectious bacterial pathogens are particularly concerning for aquaculture and affect many economically important cultured fishes such as salmon, trout, tilapia, and catfish [8,9]. Additionally, billions of US dollars are lost annually due to bacterial diseases in aquaculture [10]. Bacterial disease outbreaks can also lead to temporary or full suspension of stocking into wild fisheries [11], potentially impacting both populations of conservation concern and recreational fisheries.

One of the most problematic salmonid pathogens in the world is the bacterium *Flavobacterium psychrophilum* that causes bacterial coldwater disease (BCWD) also known as rainbow trout fry syndrome (RTFS) in many European countries. Bacterial coldwater disease is characterized by a white discoloration around the adipose fin at the early stages of the disease that transitions into caudal peduncle lesions, revealing underlying muscles and bones [12,13]. Epizootics of BCWD typically occur at low water temperatures, between 4° and 10 °C [14], accounting for the name bacterial coldwater disease; however, outbreaks have been reported in water temperatures up to 15 °C [15]. Outbreaks have also been reported in different species of salmonids since 1948 [14,16]. *Flavobacterium psychrophilum* infections typically affect age-0 salmonids but can occur in older individuals [12,17,18]. Mortality associated with infection can be up to 90% in hatchery settings [17,19] depending on temperature and developmental stage of the host [20,21]. 

Laboratory exposure studies are typically conducted to evaluate mortality, disease severity, transmission, and vaccine efficacy. Mortality associated with bacterial exposure is a commonly reported and relevant endpoint. Other commonly reported experimental factors associated with BCWD mortality include bacteria culture method, bacteria culture time, exposure method, bacterial isolate, vaccine type, host immunity, fish species such as rainbow trout (*Oncorhynchus mykiss*) strains with genetic resistance to *F. psychrophilum*, and fish size [22,23,24,25,26,27,28,29,30]. However, generalizing from published studies of mortality is difficult because of variability in experimental factors among studies. For instance, mortality due to bacterial exposure has been shown to be dose dependent [31,32] but the bacterial doses differ among challenge experiments [27,32,33,34]. Similarly, differences in culture time have been shown to be dependent on the length of time bacteria are cultured [22]. Typical bacterial culture times range between 18 h and 96 h [22,23,31,32,35]. Bacterial coldwater disease can affect larger individuals, as well as smaller age-0 fish, and creates high mortality [14,32], suggesting that fish size is also an important factor. The method used to expose fish to the bacterium may also play a role in mortality differences observed among studies. The most common exposure methods are intramuscular injection, intraperitoneal injection, subcutaneous injections, and bath immersion. Comparisons among exposure methods illustrate that intramuscular injections resulted in higher fish mortality than intraperitoneal injections [31]. Due to the worldwide distribution of *F. psychrophilum*, there are many isolates that vary in pathogenicity and host species [36,37,38,39]. However, due to variation among laboratories, protocols, and experimental goals, it is difficult to make general inferences regarding the relationship between experimental factors and disease outcomes. 

Here, we review the *F. psychrophilum* literature to investigate factors affecting variation in mortality in susceptible rainbow trout during experimental exposures to *F. psychrophilum*. In particular, we examined how bacterial dose, culture time, exposure method, bacterial isolate, number of challenge days, bath exposure time, exposure media, and mass of the fish impacted mortality of rainbow trout. In addition, we used Bayesian analytical methods to compare how different protocols affect mortality due to *F. psychrophilum* exposure. Understanding how differences in exposure protocols affect exposure outcomes, like mortality may support advances in knowledge and management of BCWD and the health of cultured salmonids around the world.

## 2. Results

### 2.1. Data Collection

Using our search criteria, 604 manuscripts met one criterion or more and 23 met all search criteria. All 23 studies from which we took data were published since 1999 (Table 1). The 23 manuscripts accounted for 142 data points that were used in the analysis. Of the 142 total data points, 43 data points were mock exposure/no treatment controls (injection or immersion exposure with no bacteria; dose and culture time equal to zero) and 99 were bacterial exposures. Rainbow trout weights reported are mean weights and ranged between 0.3 g and 300 g. Ten different bacterial isolates were identified (Table 1). Bacterial doses ranged from 7.00 × 10^2^ colony forming units per mL (CFU/mL) to 8.90 × 10^8^ CFU/mL. Four methods of exposures (mock exposures and bacterial exposures) were documented, including subcutaneous injection (*n* = 42 data points), intraperitoneal injection (*n* = 60), intramuscular injection (*n* = 22) and bath immersion exposure (*n* = 18). When bath immersion was used, time of exposure ranged between 1 h and 48 h. The number of challenge days ranged between 6 days and 32 days. The duration the bacterium was cultured ranged between 18 and 72 h (Table 1).

### 2.2. Regression

**Figure 1:**
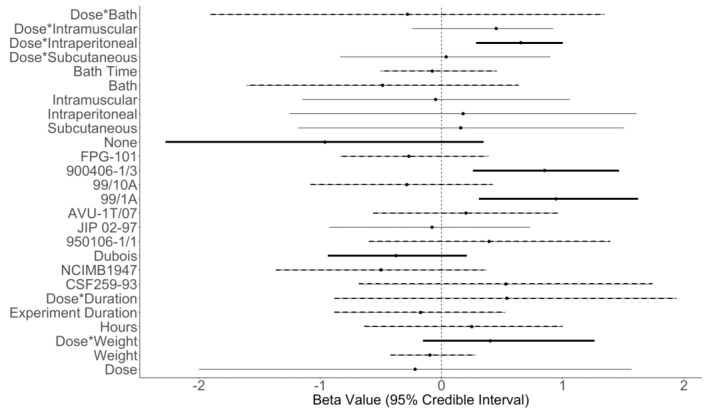
Calculated regression coefficients (x-axis) for each covariate (y-axis), including dose (CFU/mL), weight (g), Dose*Weight, hours, isolate (CSF259-93 through FPG-101), exposure type (none [Control or mock injection]; subcutaneous, intraperitoneal, or intramuscular injection; bath), and Dose*Exposure type. Black dots represent the mean and the horizontal lines represent the 95% credible intervals. Vertical dotted black line denotes zero. Black horizontal lines indicate the probability of the regression coefficient being negative or positive for their respective direction (thick black lines indicate over 90% probability, dashed black lines indicate between 61–89% probability, thin black lines indicate between 40–60% probability).

The two Markov chain Monte Carlo (MCMC) chains mixed and converged to the target distributions (visual inspection of trace plots and Gelman and Rubin diagnostic values = 1.0 for all model parameters) and the model fit the data well (Bayesian *p*-value for the expected value of mortality = 0.64; Bayesian *p*-value for the standard deviation for the expected value of mortality = 0.28). The standard deviation of the mortality values among the data was 0.04. To account for the non-independence of multiple data points from the same manuscript, we included an intercept term for each manuscript. Intercepts ranged between −1.25 and −0.11 and all manuscripts had a mean negative intercept with large overlapping 95% credible intervals, indicating that the majority of studies that we included did not have some large random effect influencing mortality (Supplementary Figure S1). Regression coefficients ranged between −0.96 and 0.94 (Table 2; Figure 1) and many coefficients had 95% credible intervals that overlapped zero or had only slight positive or negative effects. Standard deviation of the regression coefficients ranged between 0.18 and 0.91, with the coefficient associated with dose having the largest variation (Table 2). The probability of mortality ranged between 0.5% and 97.27% across all data. The multiplicative change in the odds of mortality based on each covariate ranged between 0.38 and 2.57 (Table 2). 

The probability of mortality increased with bacterial dose through interactions with weight (Dose*Weight), experimental duration (Dose*Duration) and exposure method, (specifically Dose*Intraperitoneal injection and Dose*Intramuscular injection but not Dose*Subcutaneous injection models). The interaction between dose and intraperitoneal injection (Dose*Intraperitoneal) was the only exposure type effect with 95% credible intervals that did not overlap zero and has a 99.9% probability of having higher mortality indicating an increase in mortality when intraperitoneal injection was used as the exposure method. Dose*Intraperitoneal injection also had the least variability of the effects that involved exposure type, with the smallest 95% credible intervals (Figure 1). The Dose*Weight interaction also showed a probability of being in the positive direction greater than 92% (Figure 1; Table 2) despite the 95% credible interval overlapping zero. The coefficients for intraperitoneal injection and Dose*Subcutaneous injection showed a slight positive effect on mortality, with probabilities ranging between 54% and 60% for the positive direction (Figure 1; Table 2). Bath exposures produced lower mortality than other exposure types (Figure 1). Of the four exposure types, bath immersion exposures also showed the lowest multiplicative change in the odds (Table 2). The controls (denoted as None; Figure 1) had the largest mean negative effect within the analysis, indicating that this group had the lowest reported mortality of the exposure types.

Comparison of the ten *F. psychrophilum* isolates included within the analysis showed that five isolates had reduced mortality (negative effects) and five isolates had increased mortality (positive effects) (Figure 1). Eight isolates had 95% credible intervals that overlapped zero, and all ten isolates had some amount of 95% credible intervals that overlapped each other. Isolates 900406-1/3 and 99/1A clearly increased the probability of mortality relative to the other isolates shown by large mean positive effects and over 99% probability of having a positive effect (Figure 1; Table 2). Isolates AVU-1T/07, 950106-1/1, and CSF259-93 also increased the probability of mortality (71–81%), indicating a minimum of 71% of the time there was a positive effect on mortality for those isolates even though their 95% credible intervals overlapped zero (Figure 1; Table 2). The five isolates that had a negative effect on mortality had a relatively small mean negative effect but probabilities of having a negative effect were 57% or greater (Table 2). Despite some overlap of the 95% credible intervals (e.g., 900406-1/3 versus 99/1A or NCIMB1947 versus JIP 02-97), we found some evidence that several bacterial isolates produce higher mortality than others (FPG-101 versus 900406-1/3, FPG-101 versus 99/1A, 900406-1/3 versus 99/10A, 99/1A versus 99/10A, 900406-1/3 versus JIP 02-97, 99/1A versus JIP 02-97, 900406-1/3 versus NCIMB1947, and 99/1A versus NCIMB1947; Figure 1). The largest overlap among these comparisons is 19.50% (900406-1/3 versus JIP 02-97), indicating that 80.5% of the time there is a difference between these two isolates.

## 3. Discussion

Our quantitative comparison of published studies describing factors associated with mortality due to *F. psychrophilum* exposure is the first to comprehensively evaluate differences among studies. Variability in mortality among published studies was high but overlapped considerably and did not appear to be affected by our selection criteria for inclusion in our analysis. Our comparison analysis also indicated that differences in mortality among studies can be attributed to the Dose*Weight interaction, Dose*Intraperitoneal injection interaction, exposure method, and bacterial isolate.

Variability in mortality associated with *F. psychrophilum* challenge among published studies occurred despite recent advances in bacterial culture methods and individual laboratory standardization of experimental methods. Our analysis indicates considerable differences in *F. psychrophilum* infection protocol among laboratories that make broader inferences regarding *F. psychrophilum* exposure among studies difficult. However, the published literature does indicate that dose, exposure method, experiment duration, and bacterial isolate are key factors to consider when designing exposure experiments. Nevertheless, those common key factors do not explain all the variation across the experiments we examined. There are other factors (e.g., water temperature, fish density, husbandry practices) that could have affected variation in mortality among the experiments. Another source of variation that we did not include within this analysis is rainbow trout strain which may be important because there are known genetic differences among trout strains [53] and known heritable variation in resistance [28,44,54,55] that could explain variable responses to bacterial exposure.

A surprising result of our analysis was the positive effect of fish weight on mortality, showing that, for the same dose, as fish weight increases so do the odds of mortality. The effect of Dose*Weight contradicts other observations in reviews and challenge experiments, which generally show that smaller age-0 fish have higher mortality than larger fish [14,20,32]. One potential reason for the difference between our results and previous findings could be the broad size range of fish used in the trials we analyzed (0.3–28 g, and 300 g) whereas most studies are focused on characterizing BCWD infection in relatively small fish (0.3–10 g). Reports in the literature do not represent all potential size ranges of rainbow trout exposed to *F. psychrophilum* which may bias the effect that weight has on mortality associated with *F. psychrophilum* infection. Another reason for the discrepancy could be a non-linear effect of weight in which mortality initially increases for age-0 fish and declines after fish reach a larger size. Investigation of non-linear effects would require a broader range of fish weights than are currently reported in the literature. Future experiments conducted with a broad range of weights would allow more thorough evaluation of the effects of size and age on mortality associated with BCWD.

Our results indicated that experimental challenge exposure of rainbow trout to *F. psychrophilum* by injection produces more mortality than by bath immersion and our findings agree with other studies suggesting that bath immersion is highly variable or leads to unreproducible results [31,32,46,56]. One potential reason for higher mortality is that injection exposures allow the bacteria to bypass the external immune responses of the fish (e.g., scales, skin, mucus) compared to bath immersion exposure, which has also been mentioned by others [31,56]. However, bath exposure was unevenly represented in the literature where only 12 of the 142 cases of mortality included bath exposure making it difficult to draw conclusions based on these comparisons. Additionally, within those 12 data points, 10 were represented by the NCIMB1947 isolate, which is considered weakly virulent [51] and could have affected the direction and magnitude of the effect of bath immersion. It appears that intraperitoneal injection leads to more mortality compared to intramuscular or subcutaneous injection based on the Dose*Intraperitoneal interaction. Given the smaller variation in mortality associated with intraperitoneal injection shown in our analysis, intraperitoneal injection may be preferable if enhanced reproducibility for the relationship between exposure dose and mortality is needed (relative to intramuscular or subcutaneous injections) and disease progression with respect to natural immune defense barriers, as would be obtained from bath exposure, is not of interest. Provided proper administration protocols are followed, injections do not damage organs, and injection controls are included, intraperitoneal injection should result in more consistent mortality during *F. psychrophilum* challenge experiments given that the goal is to assess bacteria-caused mortality and not immune defense barriers, i.e., not mortality from the exposure process itself. More consistent protocols and results may lead to easier comparison between experiments involving *F. psychrophilum* exposure.

Our analysis suggests that bacterial isolates differ in their effect on mortality, with some producing higher mortality compared to others, similar to other studies [51,57]. Our analysis provides a formal way of comparing and viewing effect sizes. The benefit of using a Bayesian approach is that it allows comparison of statistically normalized distributions and calculation of the probability of an effect being positive or negative [58]. Comparisons between some isolates indicated a relatively high probability of differing in their effect on mortality, while others did not seem to differ in their effects on fish mortality. Some isolates, such as FPG-101 and 99/10A as well as Dubois, have 100% overlap indicating no difference in mortality between the isolates. Despite the overlap observed for some isolate pairs there are several comparisons between isolates that show large differences in mortality; for example, 900406-1/3 and JIP 02-97 showed an 80.5% probability of producing differences in mortality. Differences in virulence and mortality among *F. psychrophilum* isolates have also been shown within the literature [26,36,37,38,39,51]. Additionally, studies show that *F. psychrophilum* has high genetic heterogeneity [37,39,59,60] and genetic variation among isolates may be connected to variation in virulence [39] which may explain the variation in mortality attributed to different isolates. It also seems likely that unique isolates would affect rainbow trout populations differentially. In vivo experiments have shown mixed results regarding host preference when using injection as exposure method [61,62,63,64]. However, Knupp et al. [62] showed high mortality for coho salmon from an isolate recovered from coho salmon and low mortality to coho salmon from an isolate recovered from rainbow trout suggesting host-specific preferences among isolates. 

Within our analysis, culture time was unevenly represented in the literature but appeared to have no effect on mortality. A total of 82 data points represented culture times of 48 h or greater (44, 48-h; 2, 66-h; 33, 72-h; 3, 96-h). Only 20 data points represented culture times ranging between 18 and 36 h (4, 18-h; 5, 24-h; 11, 36-h) with the remaining 30 data points representing no treatment controls with zero culture time. One explanation for higher culture time reported in the literature is the potential for maximizing numbers of bacteria harvested and an overall higher concentration of bacteria for experimental use. However, as culture time increases, resources in the culture media decrease, leading to a combination of bacteria that are actively replicating and inactive bacteria that are in a stationary or non-growth phase [65]. A potential reason for culture time spanning both negative and positive values, and thereby showing variable effects on mortality, is that bacteria in the stationary growth phase may have lower virulence. Kondo et al. [66] suggested that *F. psychrophilum* harvested at the logarithmic phase (the maximum population growth rate of the bacteria) may result in higher virulence, and Aoki et al. [22] showed that bacteria cultured at 18 and 24 h resulted in greater fish mortality than bacteria cultured at 48 h. Our compilation of the collected data suggests that the current *F. psychrophilum* published literature is lacking in experimental infections when *F. psychrophilum* were harvested during their logarithmic growth phase, ranging between 18 and 36 h of culture time [16,22]. Bacteria harvested during the logarithmic growth phase may provide more consistent fish mortality rates because the bacteria are all actively replicating. Defining the growth phase of the bacteria as well as the culture times of within-challenge experiments may help researchers better associate bacterial growth phase with mortality in the fish and allow replication in future challenge experiments.

Bacterial dose is an important aspect of *F. psychrophilum* exposure experiments. Mortality has been shown to be dose dependent [31,32,67] indicating that dose needs to be accounted for in exposure experiments. Our comparison analysis suggested that dose by itself has no effect on mortality and is highly variable. However, the increase in mortality due to the Dose*Weight and Dose*Intraperitoneal injection interactions indicate that dose does affect mortality due to *F. psychrophilum* exposure. Mortality based on dose is expected to be variable because of the differences in isolates, exposure methods, and different fish weights. Because there is variable mortality based on bacterial dose, pilot experiments [67] may provide necessary dose-specific information until standardized procedures are achieved within the laboratory.

For data to be included in the analysis, manuscripts had to state, clearly and explicitly, experimental factors used and to report the factors with respect to fish mortality and experimental unit without our making assumptions as to what was done. As such, our analysis did not include all possible factors that may affect mortality in an *F. psychrophilum* challenge experiment. For instance, the effect of culturing the bacteria on agar versus in broth on mortality was not included within the analysis. Additional factors that may affect mortality include fish age, aquarium system design, prestress treatments, growth media, fish density within experimental tanks, ingredients of bacterial suspension, inoculation media, husbandry practices, feeding rates, feed type, water temperature, host species, host strain, and others. A number of factors were not reported, and assumption of experimental values would have led to incorrect data incorporation across manuscripts. This resulted in manuscripts/data not being included within the analysis because assuming data values associated with experimental factors could lead to biased results. Examples of experimental factor values that were not explicitly stated included providing ranges of values (e.g., temperatures, feeding rates, tank sizes) and not reporting certain factors (e.g., inoculation media, age of fish, aquarium/water system type, feed rates). 

Even the focal studies that explicitly reported experimental factors varied across the literature, possibly due to the wide range of research goals and writing styles. Only 12% of the focal studies reported fish age, compared to 100% of the manuscripts reporting average fish weight. Regardless, age and weight are correlated in fish so would not be used together in a statistical model. While an association with low temperature has been reported for BCWD infections [14,15], mean time to death is shortest in water temperatures between 12–15 °C [68]. All manuscripts included within the analysis reported water temperatures ranging from 10–17 °C, indicating that temperature is an important experimental factor. However, some manuscripts, for example Wagner and Oplinger [44] and Schubiger et al. [45], reported a range in water temperatures but had no explicit value linked to experimental mortality. Rainbow trout was the only species used in our analysis to reduce model complexity. Rainbow trout strain was not included in our analysis because it was not commonly reported, however, strain may explain some of the variability in mortality that is seen among isolates. In the presence of selection, different rainbow trout populations have shown resistance to *F. psychrophilum* [26,30,54]. Similar disease dynamics have been observed with variable susceptibility to whirling disease among rainbow trout strains [69,70]. If future manuscripts focused on *F. psychrophilum* challenge experiments explicitly state all possible factors that could be associated with mortality, standardization and reproducibility in future work would be facilitated.

Our analysis allowed for formal comparison of experimental factors across the literature and inference on the factors affecting rainbow trout mortality due to exposure to *F. psychrophilum* and suggests avenues to further standardize research protocols and goals. Establishment of protocols that will result in comparable outcomes across laboratories, provide the ability to use prior experimental information in future experiments, and improve the ability to evaluate management options to mitigate *F. psychrophilum* infection will all support advancement of our understanding of the effects of BCWD on the health of cultured salmonids. Moving forward, our results provide steppingstones for reducing variation in *F. psychrophilum* mortality and, in turn, sets the stage for more powerful inferences and better comparisons across laboratories.

## 4. Materials and Methods

### 4.1. Data Collection

We identified studies published in peer reviewed journals in Web of Science, Academic Search Premier, and Google Scholar using combinations of search terms that are associated with bacterial coldwater disease, rainbow trout fry syndrome, and *F. psychrophilum* research (Table 3). The term “bacterial coldwater disease” covered and reported other spellings such as: “bacterial cold water disease” and “bacterial cold-water disease” within the search engines. We also identified studies by searching the literature cited sections of papers obtained during the initial search. Literature search occurred in November 2020. Due to advancements in reproducibility of culture methods and challenge standardization occurring around 1999 [16], manuscript dates were restricted from 1999 to 2020. The previous scientific names (*Cytophaga psychrophilla* and *Flexibacter psychrophilus*) were not used because the name changed to *Flavobacterium psychrophilum* in the mid to late 1990s [71]. Due to the broad variety of research goals, wide ranging experimental factors, and statistical requirements, not all published studies were included within our dataset. We only included studies that used rainbow trout as the study species and that reported the *F. psychrophilum* isolate used, number of hours the bacterium was cultured, infection/exposure method, number of challenge days, exposure media, amount of time associated with exposure method (time for bath exposure), bacterial dosage used, weight of fish at the time of infection, and mortality or the number of fish dead out of the total number of fish infected. We extracted data manually from the text, tables, figures, and provided supplementary information, taking only information pertaining to susceptible (able to contract BCWD/RTFS) and naïve (never exposed to *F. psychrophilum*) rainbow trout within *F. psychrophilum* experiments. Each data point in our analysis consisted of the dependent variable, mortality (defined as cumulative percent mortality which was constructed from reported percent mortality, percent survival, cumulative percent mortality, and/or change in number of fish from a starting value), and associated covariates thought to affect mortality including dose used to expose the fish (mock exposure/no treatment controls represented by zero dose), weight of the fish, hours the bacteria were cultured (mock exposure/no treatment controls represented by zero hours), exposure method (intraperitoneal injection, subcutaneous injection, intramuscular injection, bath immersion), amount of time for bath exposures, number of days the challenge experiment lasted, bacterial isolate, the interaction between dose and fish weight (Dose*Weight), the interaction between dose and number of days the challenge experiment lasted (Dose*Duration), and the interaction between dose and the exposure method (Dose*exposure method). 

### 4.2. Regression

Prior to statistical modeling of mortality, all covariate pairs were examined for collinearity. Each non-numerical categorical variable (e.g., bacterial isolate) was transformed into an indicator variable, represented by a zero or one. Due to large differences in units and scale for each predictor, we standardized all covariate values prior to analysis by subtracting the mean of the covariate group and dividing by two standard deviations [72] (Supplementary Table S1). 

Mortality, measured as the number of dead fish (*y_i,j_*), was analyzed using a hierarchical Bayesian binomial beta regression model (bold indicates vectors):

(1) $$g\left(S_{0,j},\;S,x_{i,j}\right)=\mathrm{inverse}\;\mathrm{logit}(S_{0,j},+S_{1}x_{i,j}+\cdots +S_{26}x_{i,j}),$$

$$\left[p,S,\;S_{0},\sigma^{2},\mu_{\zeta },\;\sigma_{\zeta }|\;y\right]\;\begin{array}{c} \propto \overset{n}{\underset{i=1}{\prod }}\overset{J}{\underset{j=1}{\prod }}\mathrm{binomial}\;(y_{i,j}\;|\;p_{i,j})\times \;\mathrm{beta}(p_{i,j}\;|\;g\left(S_{0,j},\;S,\;x_{i,j}\right),\;\sigma^{2})\\ \times \mathrm{normal}(S_{0,j}\;|\;\mu_{\zeta },\;\sigma_{\zeta })\times \mathrm{normal}(S\;|\;0,\;1.96)\times \mathrm{uniform}(\sigma^{2}\;|\;0,\;100)\\ \times \mathrm{normal}(\mu_{\zeta }\;|\;0,\;1.96)\times \mathrm{uniform}(\sigma_{\zeta }\;|\;0,\;100)\; \end{array}$$

using Just Another Gibbs Sampler (JAGS) within program R (version 4.1.3) [73]. The *i* represents observation, *j* represents the individual manuscripts, *p_i,j_* represents the probability of mortality, *S*_0,*j*_ represents the intercept that varies with manuscript accounting for the non-independence of data from the same manuscript of how the data were collected, and *S*_1,…,26_ represents regression coefficients associated with each covariate (see Table S1 for full listing of the covariates considered). The use of a binomial beta mixture distribution allowed for added variation not accounted for with a traditional binomial regression model. Vague prior information was used for each regression coefficient parameter (*S*) associated with each covariate and the mean of the intercepts (*μ_ξ_*), with the means equal to zero and the variance equal to 1.96 from a normal distribution. Because we used a logit link function in the model, the variance was set at 1.96 [73]. Vague prior information for variances (*σ* and *σ_ξ_*,) was from a uniform distribution ranging from zero to 100. 

Posterior inference for model parameters and derived quantities were based on three chains of 680,000 Markov chain Monte Carlo (MCMC) samples following convergence after a burn in period of 20% of the total iterations (170,000). Convergence for model parameters was determined by visual inspection of mixed trace plots and the use of Gelman and Rubin diagnostic values [74]. Gelman and Rubin diagnostic values less than 1.1 indicate model convergence [58]. Inferences about mortality were evaluated by examining the posterior distribution for each covariate allowing for a calculated mean and 95% credible interval for each factor. Lack of fit was assessed using posterior predictive checks, or Bayesian *p*-values, where values <0.1 and >0.9 indicate lack of fit [58]. We present the multiplicative change in odds of mortality (e^regression coefficient^), mean effect size, and the associated 95% credible intervals for each covariate to illustrate their impact on fish mortality. 

## Figures and Tables

**Table 1:** Data used within the analysis showing how many data points were included, *F. psychrophilum* dose range (CFU/mL), exposure type (subcutaneous injection (Sub), intramuscular injection (IM), intraperitoneal injection (IP), and bath immersion), experiment duration (days), bacterial isolate used, weight range (g), number of culture hours used, and length of bath immersion (hours) from each author.

Data Points	Dose Range	Exposure Type	Experiment Duration	Isolate	Weight (g)	Culture Hours	Bath Time	Manuscript	Author
31	700–4.0 × 10^7^	IP	28	950106-1/1, 99/1A, 99/10A, 900406-1/3	1.0–28	48	NA	1	[32]
11	4.2 × 10^3^–4.2 × 10^7^	IP, IM	29	JIP 02-97	4.5	36	NA	2	[31]
6	1.0 × 10^6^	IP	6	Dubois	1–300	72	NA	3	[20]
12	1.25 × 10^4^–1.25 × 10^6^	Sub	28	CSF259-93	1.4	72	NA	4	[27]
3	6.25 × 10^6^–6.25 × 10^7^	Sub	28	CSF259-93	7.5	72	NA	5	[40]
10	2.0 × 10^6^–3.4 × 10^8^	Bath	14	NCIMB1947	1.1–6.4	18–66	1	6	[22]
6	6.25 × 10^6^–6.25 × 10^7^	Sub	28	CSF259-93	4.6, 15	48	NA	7	[33]
8	3.0 × 10^5^–7.0 × 10^6^	Sub	28	CSF259-93	5	72	NA	8	[41]
2	1.0 × 10^5^–1.0 × 10^7^	Bath	30	950106-1/1	0.77	24–48	24, 48	9	[42]
3	2.0 × 10^8^–5.0 × 10^8^	Sub	28	CSF259-93	2.3	72	NA	10	[43]
6	5.0 × 10^7^–1.20 × 10^8^	Sub	28	CSF259-93	5, 10	96	NA	11	[35]
4	7.9 × 10^8^–8.9 × 10^8^	Bath	28	CSF259-93	0.3, 1.5	48	1	12	[34]
6	3.6 × 10^6^–6.3 × 10^7^	IP	21	CSF259-93	6.8–9.5	72	NA	13	[44]
2	1.03 × 10^7^	Sub	28	CSF259-93	4	72	NA	14	[45]
2	1.2 × 10^7^	IP	28	CSF259-93	0.5	72	NA	15	[46]
4	3.03 × 10^7^	IP	21	FPG-101	7.5	72	NA	16	[47]
4	2.29 × 10^7^–3.0 × 10^7^	IP	28	CSF259-93	3.08, 4.27	72	NA	17	[48]
2	1.0 × 10^8^	Bath	28	AVU-1T/07	12.46, 12.75	24	5	18	[49]
2	1.4 × 10^7^	IP	28	950106-1/1	3.5	48	NA	19	[24]
1	4.7 × 10^7^	IM	28	CSF259-93	3.5	72	NA	20	[50]
12	1 × 10^3^–1 × 10^7^	IM	21	NCIMB1947, 950106-1/1	5	48	NA	21	[51]
2	1.0 × 10^6^	Sub	28	CSF259-93	3.5	72	NA	22	[52]
3	1.34 × 10^5^	IM	21	CSF259-93	1.4–11.3	18–72	NA	23	[23]

**Table 2:** Posterior regression coefficients (S) from standardized data with mean values, standard deviation values, the represented multiplicative change and the probability of the regression coefficient being negative or positive for dose (CFU/mL), weight (g), Dose*Weight, hours, isolate (CSF259-93 through FPG-101), exposure type (none [Control or mock injection]; subcutaneous, intraperitoneal, or intramuscular injection; bath), and Dose*Exposure type. [Neg., Pos] represents the probability of the regression coefficient having a negative or positive effect.

Parameter	Mean	Standard Deviation	Multiplicative Change	[Neg., Pos.]
S_Dose_	−0.22	0.91	0.80	[59%, 41%]
S_Weight_	−0.10	0.18	0.91	[72%, 28%]
S_Dose*Weight_	0.40	0.33	1.50	[8%, 92%]
S_Hours_	0.25	0.41	1.28	[25%, 75%]
S_Experiment Duration_	−0.17	0.39	0.84	[69%, 31%]
S_Dose*Duration_	0.54	0.72	1.72	[23%, 77%]
S_CSF259-93_	0.53	0.61	1.70	[19%, 81%]
S_NCIMB1947_	−0.50	0.44	0.61	[87%, 13%]
S_Dubois_	−0.37	0.30	0.69	[91%, 9%]
S_950106-1/1_	0.39	0.50	1.48	[22%, 78%]
S_JIP 02-97_	−0.078	0.42	0.93	[57%, 43%]
S_AVU-1T/07_	0.20	0.38	1.22	[29%, 71%]
S_99/1A_	0.94	0.33	2.57	[0.2%, 99.8%]
S_99/10A_	−0.29	0.39	0.75	[77%, 23%]
S_900406-1/3_	0.85	0.31	234	[0.3%, 99.7]
S_FPG-101_	−0.27	0.30	0.76	[84%, 16%]
S_None_	−0.96	0.67	0.38	[93%, 7%]
S_Subcutaneous_	0.16	0.69	1.17	[40%, 60%]
S_Intraperitoneal_	0.18	0.73	1.20	[40%, 60%]
S_Intramuscular_	0.048	0.56	0.95	[54%, 46%]
S_Bath_	−0.49	0.57	0.62	[81%, 19%]
S_Bath Time_	−0.076	0.25	0.93	[66%, 34%]
S_Dose*Subcutaneous_	0.039	0.44	1.04	[46%, 54%]
S_Dose*Intraperitoneal_	0.65	0.18	1.92	[0.1%, 99.9%]
S_Dose*Intramuscular_	0.45	0.28	1.57	[7%, 93%]
S_Dose*Bath_	−0.28	0.83	0.76	[65%, 35%]

**Table 3:** The search terms and Boolean terms used for data collection in Web of Science, Academic Search Premier, and Google Scholar.

	Search Terms
1	*Flavobacterium psychrophilum*
2	bacterial coldwater disease
3	Rainbow Trout Fry Syndrome
4	*Flavobacterium psychrophilum* AND challenge
5	bacterial coldwater disease AND challenge
6	Rainbow Trout Fry Syndrome AND challenge
7	*Flavobacterium psychrophilum* AND experiment
8	bacterial coldwater disease AND experiment
9	Rainbow Trout Fry Syndrome AND experiment
10	*Flavobacterium psychrophilum* AND exposure
11	bacterial coldwater disease AND exposure
12	Rainbow Trout Fry Syndrome AND exposure
13	*Flavobacterium psychrophilum* AND infection
14	bacterial coldwater disease AND infection
15	Rainbow Trout Fry Syndrome AND infection
16	*Flavobacterium psychrophilum* AND challenge OR experiment OR exposure OR infection
17	bacterial coldwater disease AND challenge OR experiment OR exposure OR infection
18	Rainbow Trout Fry Syndrome AND challenge OR experiment OR exposure OR infection
19	*Flavobacterium psychrophilum* OR bacterial coldwater disease OR Rainbow Trout Fry Syndrome AND challenge OR experiment OR exposure OR infection

## Data Availability

At the time of publication, data were not publicly available from Colorado Parks and Wildlife or Colorado State University. Please contact author (BWA).

## Supplementary Materials

The following supporting information can be downloaded at: https://www.mdpi.com/article/10.3390/pathogens11111318/s1, Table S1. Factors affecting post-challenge survival of *Flavobacterium psychrophilum* in susceptible rainbow trout from the literature; Figure S1. Factors affecting post-challenge survival of Flavobacterium psychrophilum in susceptible rainbow trout from the literature.

## References

[1] Berger L., Speare R., Daszak P., Green D.E., Cunningham A.A., Goggin C.L., Slocombe R., Ragan M.A., Hyatt A.D., McDonald K.R. (1998). Chytridiomycosis causes amphibian mortality associated with population declines in the rain forests of Australia and Central America. Proc. Natl. Acad. Sci. USA.

[2] Daszak P., Berger L., Cunningham A.A., Hyatt A.D., Green D.E., Speare R. (1999). Emerging Infectious Diseases and Amphibian Population Declines. Emerg. Infect. Dis..

[3] Muths E., Corn P.S., Pessier A.P., Green D.E. (2003). Evidence for disease-related amphibian decline in Colorado. Biol. Conserv..

[4] Robinson R.A., Lawson B., Toms M.P., Peck K.M., Kirkwood J.K., Chantrey J., Clatworthy I.R., Evans A.D., Hughes L.A., Hutchinson O.C. (2010). Emerging Infectious Disease Leads to Rapid Population Declines of Common British Birds. PLoS ONE.

[5] Skerratt L.F., Berger L., Speare R., Cashins S., McDonald K.R., Phillott A.D., Hines H.B., Kenyon N. (2007). Spread of chytridomycosis has caused the rapid global decline and extinction of frogs. Ecohealth.

[6] Edmunds D.R., Kauffman M.J., Schumaker B.A., Lindzey F.G., Cook W.E., Kreeger T.J., Grogan R.G., Cornish T.E. (2016). Chronic Wasting Disease Drives Population Decline of White-Tailed Deer. PLoS ONE.

[7] Vincent E.R. (1996). Whirling disease and wild trout: The Montana experience. Fisheries.

[8] Klesius P.H., Pridgeon J.W. Live attenuated bacterial vaccines in aquaculture. Proceedings of the 9th International Symposium on Tilapia in Aquaculture.

[9] Pridgeon J.W., Klesius P.H. (2012). Major bacterial diseases in aquaculture and their vaccine development. CAB Rev..

[10] Subasinghe R.P., Bondad-Reantaso M.G., McGladdery S.E. (2001). Aquaculture Development, Health and Wealth. Technical Proceedings of the Conference on Aquaculture in the Third Millennium Bangkok: NACA and FAO. http://www.fao.org/docrep/003/ab412e/ab412e09.htm.

[11] Piper R.G., McElwain I.B., Orme L.E., McCaren J.P., Fowler L.G., Leonard J.R. (1982). Fish Hatchery Management (No. 2175).

[12] Cipriano R.C., Holt R.A. (2005). Flavobacterium psychrophilum, Cause of Bacterial Cold-Water Disease and Rainbow Trout Fry Syndrome.

[13] LaFrentz B.R., Cain K.D. (2004). Bacterial Coldwater Disease. An Extension Bulletin for the Western Regional Aquaculture Center.

[14] Borg A.F. (1960). Studies on myxobacteria associated with diseases in salmonid fishes. J. Wildl. Dis..

[15] Rucker R.R., Earp B.J., Ordal E.J. (1954). Infectious Diseases of Pacific Salmon. Trans. Am. Fish. Soc..

[16] Michel C., Antonio D., Hedrick R.P. (1999). Production of viable cultures of *Flavobacterium psychrophilum*: Approach and control. Res. Microbiol..

[17] Barnes M.E., Brown M.L. (2011). A review of *Flavobacterium psychrophilum* biology, clinical signs, and bacterial cold water disease prevention and treatment. Open Fish Sci. J..

[18] Nicolas P., Mondot S., Achaz G., Bouchenot C., Bernardet J.-F., Duchaud E. (2008). Population Structure of the Fish-Pathogenic Bacterium *Flavobacterium psychrophilum*. Appl. Environ. Microbiol..

[19] Nilsen H., Olsen A.B., Vaagnes O., Hellberg H., Bottolfsen K., Skjelstad H., Colquhoun D.J. (2011). Systemic *Flavobacterium psychrophilum* infection in rainbow trout, *Oncorhynchus mykiss* (Walbaum), farmed in fresh and brackish water in Norway. J. Fish Dis..

[20] Decostere A., D’Haese E., Lammens M., Nelis H., Haesebrouck F. (2001). In vivo study of phagocytosis, intracellular survival and multiplication of *Flavobacterium psychrophilum* in rainbow trout, *Oncorhynchus mykiss* (Walbaum), spleen phagocytes. J. Fish Dis..

[21] Wood J.W. (1974). Diseases of Pacific Salmon: Their prevention and treatment.

[22] Aoki M., Kondo M., Kawai K., Oshima S. (2005). Experimental bath infection with *Flavobacterium psychrophilum*, inducing typical signs of rainbow trout *Oncorhynchus mykiss* fry syndrome. Dis. Aquat. Org..

[23] Bruce T.J., Ma J., Knupp Loch T.P., Faisal M., Cain K.D. (2020). Cross-protection of a live-attenuated *Flavobacterium psychrophilum* immersion vaccine against novel *Flavobacterium* spp. and *Chryseobacterium* spp. strains. J Fish Dis..

[24] Chettri J.K., Al-Jubury A., Dalsgaard I., Heegaard P.M.H., Buchmann K. (2018). Experimental anal infection of rainbow trout with *Flavobacterium psychrophilum*: A novel challenge model. J. Fish Dis..

[25] Fredriksen B.N., Furevik A., Gauthier D., Egenberg M., Paulsen E.D., Brudeseth B. (2013). Intramuscular challenge of rainbow trout (*Oncorhynchus mykiss*) with two Norwegian field strains of *Flavobacterium psychrophilum*. Fish Shellfish Immunol..

[26] Jarau M., Di Natale A., Huber P.E., MacInnes J.I., Lumsden J.S. (2018). Virulence of *Flavobacterium psychrophilum* isolates in Rainbow Trout *Oncorhynchus mykiss* (Walbaum). J. Fish Dis..

[27] LaFrentz B., LaPatra S.E., Jones G.R., Cain K.D. (2003). Passive immunization of rainbow trout, *Oncorhynchus mykiss* (Walbaum), against *Flavobacterium psychrophilum*, the causative agent of bacterial coldwater disease and rainbow trout fry syndrome. J. Fish Dis..

[28] Leeds T.D., Silverstein J.T., Weber G.M., Vallejo R.L., Palti Y., Rexroad C.E., Evenhuis J., Hadidi S., Welch T.J., Wiens G.D. (2010). Response to selection for bacterial cold water disease resistance in Rainbow Trout. J Anim Sci..

[29] Madsen L., Dalsgaard I. (2000). Comparative studies of Danish *Flavobacterium psychrophilum* isolates: Ribotypes, plasmid profiles, serotypes and virulence. J. Fish Dis..

[30] Wiens G.D., LaPatra S.E., Welch T.J., Evenhuis J.P., Rexroad C.E., Leeds T.D. (2013). On-farm performance of Rainbow Trout (*Oncorhynchus mykiss*) selectively bred for resistance to bacterial cold water disease: Effect of rearing environment on survival phenotype. Aquaculture.

[31] Garcia C., Pozet F., Michel C. (2000). Standardization of experimental infection with *Flavobacterium psychrophilum*, the agent of rainbow trout *Oncorhynchus mykiss* fry syndrome. Dis. Aquat. Org..

[32] Madsen L., Dalsgaard I. (1999). Reproducible methods for experimental infection with *Flavobacterium psychrophilum* in rainbow trout *Oncorhynchus mykiss*. Dis. Aquat. Org..

[33] LaFrentz B.R., LaPatra S.E., Call D.R., Cain K.D. (2008). Isolation of rifampicin resistant *Flavobacterium psychrophilum* strains and their potential as live attenuated vaccine candidates. Vaccine.

[34] Long A., Fehringer T.R., LaFrentz B.R., Call D.R., Cain K.D. (2014). Development of a waterborne challenge model for *Flavobacterium psychrophilum*. FEMS Microbiol. Lett..

[35] LaFrentz B.R., LaPatra S.E., Call D.R., Cain K.D. (2014). Immunization of rainbow trout *Oncorhynchus mykiss* (Walbaum) with a crude lipopolysaccharide extract from *Flavobacterium psychrophilum*. Aquac. Res..

[36] Apablaza P., Loland A.D., Brevik O.J., Ilardi P., Battaglia Nylund A. (2013). Genetic variation among *Flavobacterium psychrophilum* isolates from wild and farmed salmonids in Norway and Chile. J. Appl. Microbiol..

[37] Siekoula-Nguedia C., Blanc G., Duchaud E., Calvez S. (2012). Genetic diversity of *Flavobacterium psychrophilum* isolated from rainbow trout in France: Predominance of a clonal complex. Vet. Microbiol..

[38] Strepparava N., Nicolas P., Wahli T., Segner H., Petrini O. (2013). Molecular epidemiology of *Flavobacterium psychrophilum* from Swiss fish farms. Dis. Aquat. Org..

[39] Van Vliet D., Wiens G.D., Loch T.P., Nicolas P., Faisal M. (2016). Genetic diversity of *Flavobacterium psychrophilum* isolates from three *Oncorhynchus* spp. in the United States, as revealed by multilocus sequence typing. J. Appl. Environ. Microbiol..

[40] LaFrentz B., LaPatra S., Jones G., Cain K. (2004). Protective immunity in rainbow trout *Oncorhynchus mykiss* following immunization with distinct molecular mass fractions isolated from *Flavobacterium psychrophilum*. Dis. Aquat. Org..

[41] Burbank D.R., Shah D.H., LaPatra S.E., Fornshell G., Cain K.D. (2011). Enhanced resistance to coldwater disease following feeding of probiotic bacterial strains to rainbow trout (*Oncorhynchus mykiss*). Aquaculture.

[42] Henriksen M.M.M., Madsen L., Dalsgaard I. (2013). Effect of Hydrogen Peroxide on Immersion Challenge of Rainbow Trout Fry with *Flavobacterium psychrophilum*. PLoS ONE.

[43] Glenn R.A., Gannam A.L., LaPatra S.E. (2014). The lack of effectiveness of roemary oil on fish feed in controlling bacterial cold-water disease in Rainbow Trout. N. Am. J. Aquac..

[44] Wagner E.J., Oplinger R.W. (2014). Comparison of the Susceptibility of Four Rainbow Trout Strains to Cold-Water Disease. J. Aquat. Anim. Health.

[45] Schubiger C.B., Orfe L.H., Sudheesh P.S., Cain K.D., Shah D.H., Call D.R. (2015). Entericidin is required for probiotic treatment (Enterobacter sp. Strain C6-6) to protect trout from cold-water disease challenge. J. Appl. Environ. Microbiol..

[46] Ghosh B., Cain K.D., Nowak B.F., Bridle A.R. (2016). Microencapsulation of a putative probiotic Enterobacter species, C6-6, to protect Rainbow Trout, *Oncorhynchus mykiss* (Walbaum), against bacterial coldwater disease. J Fish Dis..

[47] Ryerse I.A., Hooft J.M., Bureau D.P., Hayes M.A., Lumsden J.S. (2016). Diets containing corn naturally contaminated with deoxynivalenol reduces the susceptibility of Rainbow Trout (*Oncorhynchus mykiss*) to experimental *Flavobacterium psychrophilum* infection. Aquac Res..

[48] Sudheesh P.S., Cain K.D. (2016). Optimization of efficacy of a live attenuated *Flavobacterium psychrophilum* immersion vaccine. Fish Shellfish Immunol..

[49] Hoare R., Ngo T.P.H., Bartie K.L., Adams A. (2017). Efficacy of a polyvalent immersion vaccine against *Flavobacterium psychrophilum* and evaluation of immune response to vaccination in Rainbow Trout fry (*Oncorhynchus mykiss* L.). Vet. Res..

[50] Ma J., Bruce T.J., Sudheesh P.S., Knupp C., Loch T.P., Faisal M., Cain K.D. (2018). Assessment of cross-protection to heterologous strains of *Flavobacterium psychrophilum* following vaccination with a live-attenuated coldwater disease immersion vaccine. J. Fish Dis..

[51] Sundell K., Landor L., Nicolas P., Jørgensen J., Castillo D., Middelboe M., Dalsgaard I., Donati V.L., Madsen L., Wiklund T. (2019). Phenotypic and Genetic Predictors of Pathogenicity and Virulence in *Flavobacterium psychrophilum*. Front. Microbiol..

[52] Ma J., Bruce T.J., Oliver L.P., Cain K.D. (2019). Co-infection of rainbow trout (*Oncorhynchus mykiss*) with infectious hematopoietic necrosis virus and *Flavobacterium psychrophilum*. J. Fish Dis..

[53] Avila B.W., Winkelman D.L., Fetherman E.R. (2022). Dual Resistance to *Flavobacterium psychrophilum* and Myxobolus cerebralis in rainbow trout (*Oncorhynchus mykiss*, Walbaum). J. Fish Dis..

[54] Silverstein J.T., Vallejo R.L., Palti Y., Leeds T.D., Rexorad I.I.I.C.E., Welch T.J., Wiens G.D., Ducrocq V. (2009). Rainbow trout resistance to bacterial cold-water disease is moderately heritable and is not adversely correlated with growth. J. Anim. Sci..

[55] Wiens G.D., Palti Y., Leeds T.D. (2018). Three generations of selective breeding improve rainbow trout (*Oncorhynchus mykiss*) disease resistance against natural challenge with *Flavobacterium psychrophilum* during early life-state rearing. Aquaculture.

[56] Decostere A., Lammens M., Haesebrouck F. (2000). Difficulties in experimental infection studies with *Flavobacterium psychrophilum* in rainbow trout (*Oncorhynchus mykiss*) using immersion, oral and anal challenges. Res. Vet. Sci..

[57] Castillo D., Donati V., Jørgensen J., Sundell K., Dalsgaard I., Madsen L., Wiklund T., Middelboe M. (2021). Comparative Genomic Analyses of *Flavobacterium psychrophilum* Isolates Reveals New Putative Genetic Determinants of Virulence Traits. Microorganisms.

[58] Hobbs N.T., Hooten M.B. (2015). Bayesian Models a Statistical Primer for Ecologists.

[59] Avendaño-Herrera R., Houel A., Irgang R., Bernardet J.-F., Godoy M., Nicolas P., Duchaud E. (2014). Introduction, expansion and coexistence of epidemic *Flavobacterium psychrophilum* lineages in Chilean fish farms. Vet. Microbiol..

[60] Nilsen H., Sundell K., Duchaud E., Nicolas P., Dalsgaard I., Madsen L., Aspán A., Jansson E., Colquhoun D.J., Wiklund T. (2014). Multilocus sequence typing identifies epidemic clones of *Flavobacterium psychrophilum* in Nordic countries. Appl. Environ. Microbiol..

[61] Ekman E., Norrgren L. (2003). Pathology and immunohistochemistry in three species of salmonids after experimental infection with *Flavobacterium psychrophilum*. J. Fish Dis..

[62] Fredriksen B.N., Furevik A., Olsen R.H., Gauthier D., Mendoza J., Norderhus E.A. (2016). Virulence of Chilean field isolates of *Flavobacterium psychrophilum* in Atlantic salmon (*Salmo salar* L.) parr. Bull Eur Assoc Fish Pathol..

[63] Knupp C., Kiupel M., Brenden T.O., Loch T.P. (2021). Host-specific preference of some *Flavobacterium psychrophilum* multilocus sequence typing genotypes determines their ability to cause bacterial coldwater disease in coho salmon (*Oncorhynchus kisutch*). J. Fish Dis..

[64] Nagai T., Nakai T. (2011). Growth of *Flavobacterium psychrophilum* in fish serum correlates with pathogenicity. J. Fish Dis..

[65] Wang L., Fan D., Chen W., Terentjev E. (2015). Bacterial growth, detachment and cell size control on polyethylene terephthalate surfaces. Sci. Rep..

[66] Kondo M., Kawai K., Yagyu K., Nakayama K., Kurohara K., Oshima S. (2001). Changes in the cell structure of *Flavobacterium psychrophilum* with length of culture. Microbiol Immunol..

[67] Avila B.W. (2021). Bacterial Coldwater Disease Investigations. Ph.D. Dissertation.

[68] Holt R.A., Amandi A., Rohovec J.S., Fryer J.L. (1989). Relation of Water Temperature to Bacterial Cold-Water Disease in Coho Salmon, Chinook Salmon, and Rainbow Trout. J. Aquat. Anim. Health.

[69] Fetherman E., Winkelman D., Schisler G., Antolin M. (2012). Genetic basis of differences in myxospore count between whirling disease-resistant and -susceptible strains of rainbow trout. Dis. Aquat. Org..

[70] Hedrick R.P., McDowell T.S., Marty G.D., Fosgate G., Mukkatira K., Myklebust K., El-Matbouli M. (2003). Susceptibility of two strains of rainbow trout (one with suspected resistance to whirling disease) to *Myxobolus cerebralis* infection. Dis. Aquat. Org..

[71] Bernardet J.F., Segers P., Vancanneyt M., Berthe F., Kersters K., Vandamme P. (1996). Cutting a Gordian knot: Emended classification and description of the genus *Flavobacterium*, emended description of the family Flavobacteriaceae, and proposal of *Flavobacterium hydatis* nom. nov. (Basonym, *Cytophaga aquatilis* Strohl and Tait 1978). Int. J. Syst. Bacteriol..

[72] Gelman A., Hill J. (2007). Data Analysis Using Regression and Multilevel/Hierarchical Models.

[73] R Core Team (2013). R: A Language and Environment for Statistical Computing.

[74] Gelman A., Rubin D.B. (1992). Inference from Iterative Simulation Using Multiple Sequences. Stat. Sci..

