# Supplementary material for: Factors Affecting Post-Challenge Survival of Flavobacterium psychrophilum in Susceptible Rainbow Trout from the Literature
Source: Pathogens. 2022 Nov 10;11(11):1318. doi: 10.3390/pathogens11111318 (PMC9692252; doi:10.3390/pathogens11111318)
Supplement: Supplementary file 1 [file pathogens-11-01318-s001.zip › pathogens-1885810-supplementary.pdf]

Factors affecting post-challenge survival of *Flavobacterium psychrophilum* in susceptible rainbow trout from the literature

Supplementary Info

Supplementary Table S1. Values used to standardize covariates used in the meta-analysis of *F. psychrophilum* experiments. Standardization provided a unitless value for each covariate that was used for comparisons across covariates in the meta-analysis. Covariates examined in the analysis included dose (CFU/mL), weight (g), Dose*Weight, hours, isolate (CSF259-93 through FPG-101), exposure type (none [Control or mock injection]; subcutaneous, intraperitoneal, or intramuscular injection; bath), and Dose*Exposure type, and were standardized by subtracting the covariate mean and dividing by two standard deviations from each data point.

Covariate Data	Mean	2*(Standard Deviation)
Dose	33531108.96	237498476
Weight	9.4767537313	72.732591605
Dose*Weight	128426577	646708166.8
Hours	41.73134328	54.42421187
Isolate: CSF259-93	0.26119403	0.881867618
Isolate: NCIMB1947	0.059701493	0.475644021
Isolate: S21	0.014925373	0.243418588
Isolate: Dubois	0.02238806	0.296994257
Isolate: 950106-1/1	0.119402985	0.650957704
Isolate: JIP 02-97	0.067164179	0.50249071
Isolate: AVU-1Y/07	0.014925373	0.243418588
Isolate: 99/1A	0.044776119	0.415176139
Isolate: 99/10A	0.044776119	0.415176139
Isolate: 900406-1/3	0.037313433	0.380479825
Isolate: FPG-101	0.014925373	0.243418588
None	0.298507463	0.918640733
Subcutaneous	0.313432836	0.931258682
Intraperitoneal	0.447761194	0.998259061
Intramuscular	0.089552239	0.573221293
Bath	0.134328358	0.684568676
Dose*Subcutaneous	8598694.03	96790480.46
Dose*Intraperitoneal	2552562.687	17310000.86
Dose*Intramuscular	1505971.642	17914526.18
Dose*Bath	20776865.67	221064624.2

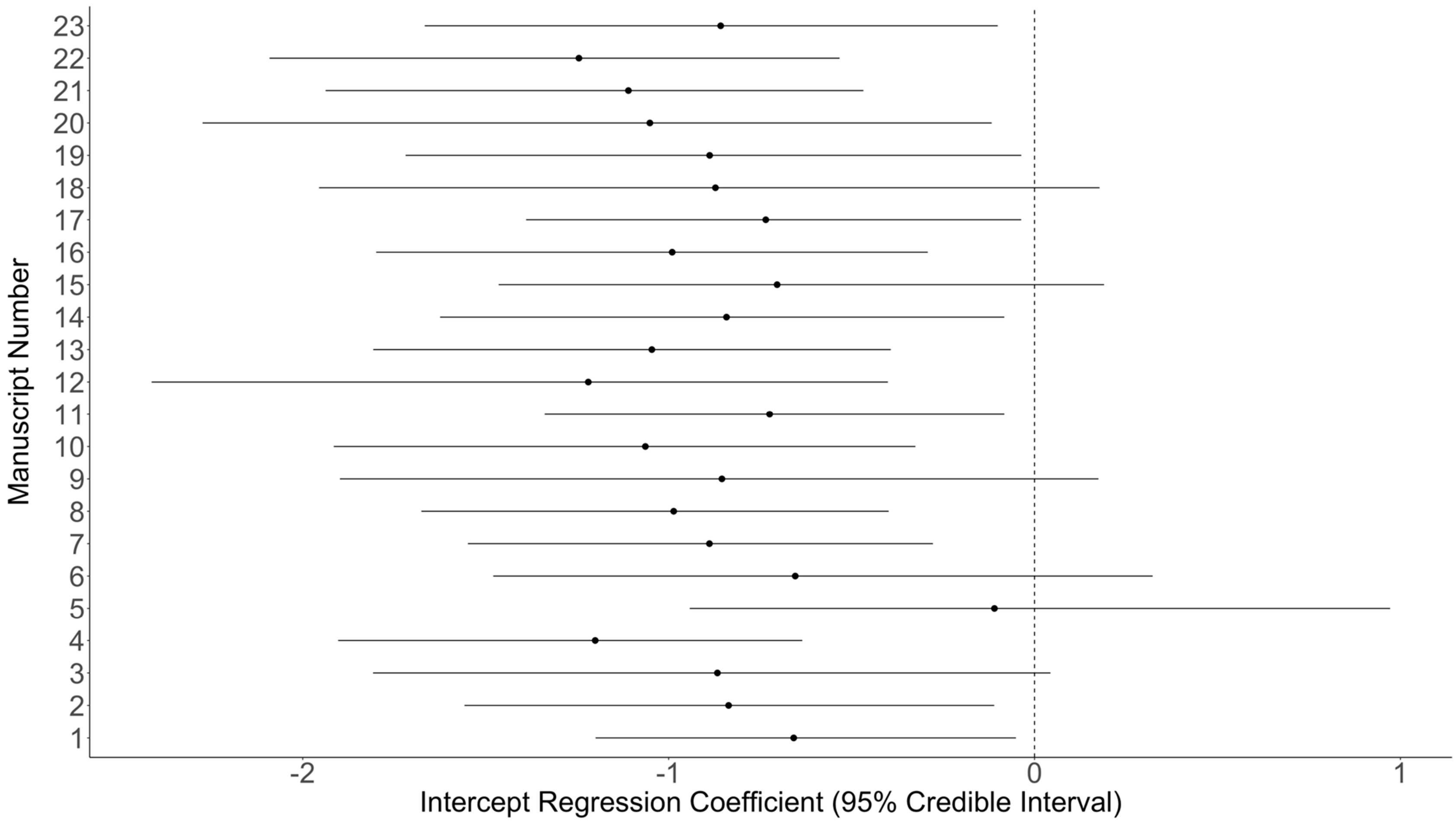

Supplementary Figure S1. Calculated intercepts (x-axis) for each manuscript (y-axis): 1) Madsen and Dalsgaard 1999, 2) Garcia et al. 2000, 3) Decostere et al. 2001, 4) LaFrentz et al. 2003, 5) LaFrentz et al. 2004, 6) Aoki et al. 2005, 7) LaFrentz et al. 2008, 8) Burbank et al. 2011, 9) Henriksen et al. 2013, 10) Glenn et al. 2014, 11) LaFrentz et al. 2014, 12) Long et al. 2014, 13) Wagner and Oplinger 2014, 14) Schubiger et al. 2015, 15) Ghosh et al. 2016, 16) Ryerse et al. 2016, 17) Sudheesh and Cain 2016, 18) Hoare et al. 2017, 19) Chettri et al. 2018, 20) Ma et al. 2018, 21) Sundell et al. 2019 22) Ma et al. 2019, and 23) Bruce et al. 2020. Black dots represent the mean and the horizontal lines represent the 95% credible intervals. Vertical dotted black line denotes zero.
